# Prevalence and Clustering of Congenital Heart Defects Among Boys With Hypospadias

**DOI:** 10.1001/jamanetworkopen.2022.24152

**Published:** 2022-07-28

**Authors:** Melissa A. Richard, Jenil Patel, Renata H. Benjamin, Emine Bircan, Stephen J. Canon, Lisa K. Marengo, Mark A. Canfield, A. J. Agopian, Philip J. Lupo, Wendy N. Nembhard

**Affiliations:** 1 Department of Pediatrics, Baylor College of Medicine, Houston, Texas; 2 Department of Epidemiology, Human Genetics, and Environmental Sciences, The University of Texas Health Science Center at Houston School of Public Health, Dallas; 3 Department of Epidemiology, Fay W. Boozman College of Public Health, University of Arkansas for Medical Sciences, Little Rock; 4 Department of Epidemiology, Human Genetics, and Environmental Sciences, The University of Texas Health Science Center at Houston School of Public Health, Houston; 5 Arkansas Children’s Hospital, Little Rock; 6 Department of Urology, University of Arkansas for Medical Sciences, Little Rock; 7 Birth Defects Epidemiology and Surveillance Branch, Texas Department of State Health Services, Austin; 8 Department of Pediatrics, University of Arkansas for Medical Sciences, Little Rock

## Abstract

**Importance:**

Hypospadias is a common birth defect of the male urinary tract that may be isolated or may co-occur with other structural malformations, including congenital heart defects (CHDs). The risk for co-occurring CHDs among boys with hypospadias remains unknown, which limits screening and genetic testing strategies.

**Objective:**

To characterize the risk of major CHDs among boys born with hypospadias.

**Design, Setting, and Participants:**

This retrospective cohort study used data from population-based birth defect surveillance programs on all male infants born in 11 US states from January 1, 1995, to December 31, 2014. Statistical analysis was performed from September 2, 2020, to March 25, 2022.

**Exposure:**

Hypospadias.

**Main Outcomes and Measures:**

Demographic and diagnostic data were obtained from 2 active state-based birth defect surveillance programs for primary analyses, the Texas Birth Defects Registry and the Arkansas Reproductive Health Monitoring System, with validation among 9 additional states in the National Birth Defects Prevention Network (NBDPN). Birth defect diagnoses were identified using the British Pediatric Association coding for hypospadias (exposure) and major CHDs (primary outcomes). Maternal covariates and birth year were also abstracted from the vital records. Poisson regression was used to estimate adjusted prevalence ratios and 95% CIs for major CHDs within Texas and Arkansas and combined using inverse variance–weighted meta-analysis. Findings were validated using the NBDPN.

**Results:**

Among 3.7 million pregnancies in Texas and Arkansas, 1485 boys had hypospadias and a co-occurring CHD. Boys with hypospadias were 5.8 times (95% CI, 5.5-6.1) more likely to have a co-occurring CHD compared with boys without hypospadias. Associations were observed for every specific CHD analyzed among boys with hypospadias, occurred outside of chromosomal anomalies, and were validated in the NBDPN. An estimated 7.024% (95% CI, 7.020%-7.028%) of boys with hypospadias in Texas and 5.503% (95% CI, 5.495%-5.511%) of boys with hypospadias in Arkansas have a co-occurring CHD. In addition, hypospadias severity and maternal race and ethnicity were independently associated with the likelihood for hypospadias to co-occur with a CHD; boys in Texas with third-degree (ie, more severe) hypospadias were 2.7 times (95% CI, 2.2-3.4) more likely than boys with first-degree hypospadias to have a co-occurring CHD, with consistent estimates in Arkansas (odds ratio, 2.7; 95% CI, 1.4-5.3), and boys with hypospadias born to Hispanic mothers in Texas were 1.5 times (95% CI, 1.3-1.8) more likely to have a co-occurring CHD than boys with hypospadias born to non-Hispanic White mothers.

**Conclusions and Relevance:**

In this cohort study, boys with hypospadias had a higher prevalence of CHDs than boys without hypospadias. These findings support the need for consideration of additional CHD screening programs for boys born with hypospadias.

## Key Points

**Question:**

What is the relative prevalence of congenital heart defects among boys born with hypospadias compared with boys born without hypospadias?

**Findings:**

In this large multistate birth defects registry cohort study of data from population-based birth defect surveillance programs on all male infants born in 11 US states, boys born with hypospadias were 6 times more likely than boys without hypospadias to have any major congenital heart defect.

**Meaning:**

This study suggests that screenings for additional birth defects among boys born with hypospadias may be warranted; in addition, molecular studies are needed to uncover the shared etiology of co-occurring defects in different developmental fields.

## Teaser

This cohort study uses data from the Texas Birth Defects Registry and Arkansas Reproductive Health Monitoring System to characterize the risk of major congenital heart defects among boys born with hypospadias.

## Introduction

Hypospadias and congenital heart defects (CHDs) are 2 of the most prevalent birth defects in the United States.^1,2^ Hypospadias is a displacement of the urethral opening in the penile shaft that affects 1 of every 125 live-born male infants,^3^ and CHDs collectively affect 1 of every 100 newborn children.^4,5^ Although the urogenital structure can be surgically corrected, individuals with hypospadias often continue to have functional difficulties,^6^ psychosocial and psychosexual dysfunction,^7,8,9,10,11^ surgical complications,^12,13^ and co-occurring health conditions beyond birth defects.^14,15^ Congenital heart defects are the leading cause of infant death due to birth defects.^16^ Approximately 25% of CHDs are critical,^17^ requiring surgical intervention in the first year of life; these critical CHDs may be detected through newborn pulse oximetry screening. Advancements in early detection and intervention have improved CHD outcomes,^18^ yet unrecognized CHD suggests that targeted screening could further reduce morbidity and mortality.

To our knowledge, few studies to date have evaluated the co-occurrence of hypospadias and CHDs.^19,20,21^ Case reports highlight key hypotheses in the shared etiology of hypospadias and CHDs (eg, midline origins),^20,22,23,24,25,26,27,28,29^ yet the exact mechanisms remain unknown. Similarly, birth defects may cluster in nonspecific patterns (ie, birth defects tend to co-occur more so than expected by chance). A recent study by Ludorf et al^21^ used an agnostic approach to characterize complex patterns of defects co-occurring with hypospadias using a large population-based birth defects registry. Congenital heart defects accounted for more than half of the most frequent defects co-occurring with hypospadias, including ostium secundum–type atrial septal defect (390 [18.7%] of 2084 cases of nonisolated hypospadias) and ventricular septal defect (290 [13.9%] of 2084 cases of nonisolated hypospadias), which lays the foundational work that hypospadias and CHDs specifically cluster.

Based on mounting evidence for the co-occurrence of hypospadias and CHDs, with advancements in CHD screening through echocardiograms, clinical examinations, and genetic sequencing, we sought to estimate the prevalence of specific CHDs among boys with hypospadias. From the specific clustering of hypospadias and CHDs established by Ludorf et al^21^ in the Texas Birth Defects Registry (TBDR), we extended these findings in independent birth defect surveillance programs. We performed a retrospective cohort study of hypospadias and CHDs among pregnancies spanning 2 decades in comprehensive birth defect surveillance programs, leveraging the strength of each program. The prevalence of CHDs in association with hypospadias was estimated between 2 contiguous states with active surveillance for major birth defects, which allows for precise estimates of CHD prevalence. Statistical validation of observed associations used data from 9 additional states throughout the US with both active and passive surveillance programs. We therefore estimated and validated the prevalence of specific CHDs that co-occur with hypospadias, leveraging data from the TBDR, the Arkansas Reproductive Health Monitoring System (ARHMS), and the National Birth Defects Prevention Network.

## Methods

This study was approved by the institutional review boards of the Texas Department of State Health Services, the University of Texas Health Science Center, and the University of Arkansas for Medical Sciences. Parental consent for all registries was waived by their respective governing bodies, based on the deidentified nature of data. We followed the Strengthening the Reporting of Observational Studies in Epidemiology (STROBE) reporting guideline for cohort studies.

### Birth Defect Registries

#### Texas Birth Defects Registry

The TBDR (N = 3 217 761 male births) conducts active surveillance of live births, stillbirths, and fetal deaths to identify infants with birth defects delivered by women residing in Texas.^30^ Statewide medical records are abstracted from all of the hospitals and delivery centers to identify chromosomal anomalies or structural birth defects that are diagnosed in the first year of life. Demographic information on the mother and child was derived from medical records and vital records obtained through the Texas Department of State Health Services. Texas Birth Defects Registry analyses included births from January 1, 1999, to December 31, 2014.

#### Arkansas Reproductive Health Monitoring System

The ARHMS (N = 488 099 male births) is an active, statewide surveillance program for birth defects conducted at Arkansas Children’s Hospital.^31^ Information on birth defects is obtained through medical record abstraction at more than 40 delivering hospitals, prenatal diagnostic clinics, and specialty clinics or hospitals. Birth defects reported in the ARHMS are diagnosed in the first 2 years of life; analyses included resident births from January 1, 1995, to December 31, 2013.

#### National Birth Defects Prevention Network

The National Birth Defects Prevention Network (NBDPN; N = 4 404 236 male births) is a network of active and passive programs from 43 birth defect surveillance registries in the US.^32^ Each surveillance program uses birth certificates to link to the index case and obtain information on demographic characteristics and births in the catchment area. Birth defects in the NBDPN are diagnosed within the first 3 years of life. Pooled individual-level data from births (1999-2007) in 9 states (Arizona, Georgia [Centers for Disease Control and Prevention], Florida, Illinois, Massachusetts, North Carolina, Nebraska, New Jersey, and New York) were available for these analyses.

### Birth Defect Classification

Guidelines developed by the National Birth Defects Prevention Study were used to classify birth defects as major or minor.^33^ Birth defects were coded by the TBDR and the ARHMS using 6-digit British Pediatric Association (BPA) codes for major birth defects. Registries in the NBDPN used BPA codes or *International Classification of Diseases, Ninth Revision* (*ICD-9*) codes, with *ICD-9* codes centrally linked to relevant BPA codes prior to analyses. From these data, we selected individuals with isolated or any co-occurring birth defects to form the following 3 groups: (1) boys with hypospadias without a CHD, (2) boys with a CHD without hypospadias, and (3) boys with co-occurring hypospadias and CHD. Hypospadias cases included all severities with or without chordee (BPA codes 752.600, 752.605-752.607, 752.620, and 752.625-752.627). Congenital heart defect cases, including critical CHDs, were grouped by major defect based on the first 4 digits of the BPA code; specific CHD codes considered minor or incompletely ascertained by the registries were excluded from analyses (eTable 1 in the Supplement). As a comparison group, we selected all boys without any major birth defects.

### Statistical Analysis

Statistical analysis was performed from September 2, 2020, to March 25, 2022. All analyses were restricted to boys. Descriptive analysis using counts and percentages was performed for demographic variables that included maternal race and ethnicity, age, and educational level. Poisson regression was used to compare the prevalence of each CHD phenotype (outcome) between boys born with hypospadias and boys born without hypospadias (exposure).^34^ All models were adjusted for maternal race and ethnicity (Hispanic, non-Hispanic Black, non-Hispanic White, and other or unknown [ie, individuals who report unknown race, multiple races, or Alaska Native, East Asian, Native American, Native Hawaiian, or South Asian ancestry]) and birth year (categorical). Regression models using NBDPN data were additionally adjusted for state of birth. Within each of the 3 data sets, regression analyses were restricted to CHDs that occurred among at least 3 boys who also had hypospadias. Regression results are reported as adjusted prevalence ratios (PRs) and 95% CIs. To generate combined PR estimates, we performed fixed-effects inverse variance–weighted meta-analysis to report overall PRs as estimated in Texas and Arkansas. Prevalence ratios across all states in the NBDPN served as validation of our primary findings from meta-analysis of the single-state registry analyses. Heterogeneity was assessed using the *I*^2^ statistic. To assess whether known syndromes were associated with the co-occurrence of hypospadias and CHDs, we performed sensitivity analyses using Texas data by excluding cases with any chromosomal syndrome (BPA codes 758.0-758.9). Analyses were conducted in R, version 3.5.2 (R Group for Statistical Computing) and Stata, version 16 (StataCorp LLC). All *P* values were from 2-sided tests and results were deemed statistically significant at *P* < .05.

We also performed analyses only among boys with hypospadias in Texas and Arkansas to characterize CHD prevalence. First, we estimated the overall prevalence and 95% CIs for any type of CHD among boys with hypospadias within each of the single-state registries. We then used logistic regression to assess the association of hypospadias severity and maternal race and ethnicity with the co-occurrence of CHD, after adjusting for birth year. Hypospadias severity was coded as first, second, or third degree, excluding hypospadias of unspecified severity from logistic regression analyses.

## Results

Among 3.7 million pregnancies in Texas and Arkansas, we identified 1485 boys who had hypospadias and a co-occurring CHD (Table 1). In both Texas and Arkansas, maternal race and ethnicity differed between hypospadias and CHD case groups. We found that boys with co-occurring hypospadias and CHD were more frequently born to non-Hispanic White mothers in both Texas and Arkansas, relative to other races and ethnicities (Texas: 41.8% of boys with hypospadias and CHD vs 35.7% of boys without hypospadias or CHD; Arkansas: 76.8% of boys with hypospadias and CHD cases vs 67.2% of boys without hypospadias or CHD). This finding was consistent with the distribution of non-Hispanic White mothers in the NBDPN states (62.7% of boys with hypospadias and CHD compared with 55.3% of boys without hypospadias or CHD; eTable 2 in the Supplement). We also observed that 17.5% of boys with hypospadias and CHD in Texas and 9.8% of boys with hypospadias and CHD in Arkansas were born to mothers aged 35 years or older at delivery (Table 1). Boys with hypospadias and CHD tended to be born to mothers with educational attainment beyond high school in Texas (45.7% of boys with hypospadias and CHD compared with 43.0% of boys without hypospadias or CHD), whereas mothers of boys with co-occurring hypospadias and CHD in Arkansas tended to have a high school education as their highest level of education (44.4% of boys with hypospadias and CHD compared with 38.4% of boys without hypospadias or CHD).

**Table 1.** Description of Maternal Traits Among Boys in the Texas Birth Defects Registry and Arkansas Reproductive Health Monitoring System

Maternal trait	Texas Birth Defects Registry: 1999-2014 (N = 3 217 761), No. (%)				Arkansas Reproductive Health Monitoring System: 1995-2013 (N = 488 099), No. (%)			
	Boys with CHDs (n = 38 691)	Boys with hypospadias (n = 17 777)	Boys with hypospadias and CHDs (n = 1343)	Boys without birth defects (n = 3 159 950)	Boys with CHDs (n = 3718)	Boys with hypospadias (n = 2846)	Boys with hypospadias and CHDs (n = 142)	Boys without birth defects (n = 481 393)
Race and ethnicity								
Hispanic	19 846 (51.3)	5559 (31.3)	576 (42.9)	1 532 438 (48.5)	336 (9.0)	91 (3.2)	8 (5.6)	34 418 (7.2)
Non-Hispanic								
Black	4072 (10.5)	2354 (13.2)	151 (11.2)	353 088 (11.2)	659 (17.7)	419 (14.7)	22 (15.5)	91 650 (19.0)
White	13 267 (34.3)	9013 (50.7)	562 (41.8)	1 128 385 (35.7)	2639 (71.0)	2288 (80.4)	109 (76.8)	323 663 (67.2)
Other^a^	1506 (3.9)	851 (4.8)	54 (4.0)	146 039 (4.6)	84 (2.3)	48 (1.7)	3 (2.1)	31 662 (6.6)
Maternal age, y								
<20	4621 (11.9)	1983 (11.2)	161 (12.0)	411 016 (13.0)	521 (14.0)	389 (13.7)	27 (19.0)	75 962 (15.8)
20-24	9628 (24.9)	4373 (24.6)	309 (23.0)	857 942 (27.1)	1142 (30.7)	894 (31.4)	41 (28.9)	160 394 (33.3)
25-29	9901 (25.6)	4898 (27.6)	360 (26.8)	860 816 (27.2)	1033 (27.8)	769 (27.0)	37 (26.1)	130 223 (27.1)
30-34	8210 (21.2)	4091 (23.0)	278 (20.7)	662 167 (21.0)	636 (17.1)	527 (18.5)	23 (16.2)	78 524 (16.3)
≥35	6324 (16.4)	2429 (13.6)	235 (17.5)	367 753 (11.7)	385 (10.4)	267 (9.4)	14 (9.8)	36 160 (7.6)
Educational level								
Less than high school	11 243 (29.1)	3321 (18.7)	328 (24.4)	887 240 (28.1)	756 (20.3)	422 (14.8)	34 (23.9)	117 861 (24.5)
High school	11 222 (29.0)	4823 (27.1)	390 (29.0)	890 660 (28.2)	1529 (41.1)	1136 (39.9)	63 (44.4)	185 087 (38.4)
More than high school	15 977 (41.3)	9532 (53.6)	614 (45.7)	1 360 239 (43.0)	1382 (37.2)	1265 (44.5)	43 (30.3)	174 096 (36.2)
Missing	249 (0.6)	101 (0.6)	11 (0.8)	21 811 (0.7)	51 (1.4)	23 (0.8)	2 (1.4)	4349 (0.9)

Abbreviation: CHDs, congenital heart defects.

^a^ Other races and ethnicities may include individuals who report unknown race, multiple races, East Asian, South Asian, Native American, Alaska Native, or Native Hawaiian ancestry.

### Prevalence of the Co-occurrence of Hypospadias and CHDs in Meta-analyses of Boys Born in Texas and Arkansas

We found that boys with hypospadias in Texas and Arkansas were 5.8 times (95% CI, 5.5-6.1) more likely to have any type of co-occurring major CHD compared with boys born without hypospadias (Table 2). Our analyses identified that critical CHDs had consistently elevated PRs across states. Based on 99 co-occurring cases in Texas and Arkansas, coarctation of the aorta was 7.5 times (95% CI, 6.2-9.2) more prevalent among boys with hypospadias. Tetralogy of Fallot was 9.3 times (95% CI, 7.4-11.7) more prevalent among boys with hypospadias than those without hypospadias, based on 80 co-occurring cases. Likewise, based on 81 co-occurring cases, transposition of the great vessels was 6.3 times (95% CI, 5.0-7.9) more likely to co-occur among boys with hypospadias. For rarer critical CHDs with fewer co-occurrences with hypospadias, we still observed elevated prevalence of the CHD in Texas, including 9.4 times (95% CI, 5.4-16.4) greater prevalence of truncus arteriosus, 6.1 times (95% CI, 4.3-8.7) greater prevalence of hypoplastic left heart syndrome, 2.8 times (95% CI, 1.2-6.8) greater prevalence of single ventricle, and 2.8 times (95% CI, 1.0-7.5) greater prevalence of Ebstein anomaly. Additional major, noncritical CHDs showed consistent PRs between Texas and Arkansas. Comparing boys with hypospadias and boys without hypospadias, atrioventricular septal defects were 5.9 times (95% CI, 4.4-7.7) more prevalent, congenital mitral stenosis was 6.7 times (95% CI, 5.1-8.7) more prevalent, and aortic valve stenosis was 5.8 times (95% CI, 4.2-7.9) more prevalent.

**Table 2.** Adjusted Prevalence Ratios for CHDs Among Boys With Hypospadias in the Texas Birth Defects Registry and Arkansas Reproductive Health Monitoring System

Type of CHD	Texas Birth Defects Registry, 1999-2014 (N = 3 217 761)^a^			Arkansas Reproductive Health Monitoring System, 1995-2013 (N = 488 099)			Inverse variance–weighted meta-analysis	
	Boys, No.		Prevalence ratio (95% CI)^b^	Boys, No.		Prevalence ratio (95% CI)^b^	Prevalence ratio (95% CI)	*I*^2^, %^c^
	With hypospadias and CHDs	With CHDs		With hypospadias and CHDs	With CHDs			
All CHDs	1343	38 691	5.8 (5.5-6.2)	142	3718	5.8 (4.9-6.9)	5.8 (5.5-6.1)	0
Bulbus cordis anomalies and anomalies of cardiac septal closure	1155	33 283	5.8 (5.5-6.2)	124	2940	6.4 (5.3-7.7)	5.9 (5.6-6.2)	0
Common truncus arteriosus^d^	13	38	9.4 (5.4-16.4)	2	20	NA	9.4 (5.4-16.4)	0
Transposition of the great vessels^d^	70	1943	6.1 (4.8-7.7)	11	187	8.0 (4.0-15.7)	6.3 (5.0-7.9)	0
Tetralogy of Fallot^d^	71	1217	9.5 (7.5-12.1)	9	162	7.1 (3.3-15.2)	9.3 (7.4-11.7)	0
Single ventricle^d^	5	327	2.8 (1.2-6.8)	0	12	NA	2.8 (1.2-6.8)	0
Ventricular septal defect	599	16 811	6.1 (5.7-6.7)	69	1619	6.7 (5.2-8.5)	6.2 (5.7-6.7)	0
Atrial septal defect	697	19 281	6.0 (5.6-6.5)	63	1361	7.1 (5.5-9.2)	6.1 (5.7-6.6)	33.4
Atrioventricular septal defect	46	1322	5.7 (4.3-7.7)	7	135	7.2 (3.2-16.4)	5.9 (4.4-7.7)	0
Other congenital anomalies of the heart	332	8891	6.2 (5.6-7.0)	34	1138	4.6 (3.2-6.5)	6.1 (5.5-6.7)	63.4
Pulmonary valve atresia or stenosis	88	2715	5.5 (4.5-6.8)	11	414	3.6 (1.8-6.9)	5.3 (4.3-6.5)	34.6
Tricuspid valve atresia or stenosis	20	555	6.1 (3.9-9.5)	2	41	NA	6.1 (3.9-9.5)	0
Ebstein anomaly^d^	<5	240	2.8 (1.0-7.5)	0	22	NA	2.8 (1.0-7.5)	0
Aortic valve stenosis	34	938	5.9 (4.2-8.3)	5	156	4.9 (2.0-11.9)	5.8 (4.2-7.9)	0
Congenital insufficiency of aortic valve	85	1764	8.0 (6.4-9.9)	0	8	NA	8.0 (6.4-9.9)	0
Congenital mitral stenosis	53	1297	6.7 (5.1-8.8)	4	97	6.6 (2.4-18.2)	6.7 (5.1-8.7)	0
Mitral valve insufficiency	242	4224	9.8 (8.6-11.2)	1	117	NA	9.8 (8.6-11.2)	0
Hypoplastic left heart syndrome^d^	32	844	6.1 (4.3-8.7)	1	129	NA	6.1 (4.3-8.7)	0
Other specified anomalies of the heart	110	3079	6.1 (5.0-7.3)	16	337	7.6 (4.5-12.8)	6.1 (5.0-7.3)	0
Unspecified anomalies of the heart	22	578	6.4 (4.2-9.9)	7	151	12.8 (5.9-28.0)	7.5 (5.2-11.0)	56
Other congenital anomalies of the circulatory system	407	8744	7.8 (7.1-8.7)	26	624	6.7 (4.5-10.0)	7.8 (7.0-8.5)	0
Coarctation of the aorta^d^	85	1935	7.3 (5.9-9.1)	14	233	9.0 (5.2-15.6)	7.5 (6.2-9.2)	0
Other anomalies of the aorta	231	4496	8.6 (7.5-9.8)	14	315	7.1 (4.1-12.2)	8.5 (7.5-9.7)	0
Anomalies of the pulmonary artery	107	2764	6.6 (5.5-8.0)	2	64	NA	6.6 (5.5-8.0)	0
Anomalies of the great veins	116	2113	9.5 (7.9-11.4)	3	146	3.4 (1.1-10.8)	9.2 (7.7-11.1)	65.1

Abbreviations: CHD, congenital heart defect; NA, not applicable.

^a^ Counts of less than 5 have been suppressed per data use agreements with the Texas Birth Defects Registry.

^b^ Poisson regression models are adjusted for maternal race and ethnicity and birth year.

^c^ Test for heterogeneity.

^d^ Critical CHD.

### Validation in the NBDPN and Sensitivity Analyses

We found that prevalence across all CHDs was elevated among boys with hypospadias in the NBDPN, consistent with our findings in Texas and Arkansas (eTable 3 in the Supplement). Although the magnitude of PR estimates varied between registry populations for some major CHDs, we identified consistent evidence that supports higher prevalence of all major CHDs among boys with hypospadias captured by the registries. Meta-analyses to combine estimates across Texas, Arkansas, and the NBDPN indicated homogeneous PRs among the critical CHDs (heterogeneity *I*^2^ < 60%), with substantial heterogeneity for other major, noncritical CHDs (heterogeneity *I*^2^ > 80%). For example, despite being a relatively common CHD, ventricular septal defects were 6.2 times (95% CI, 5.7-6.7) more prevalent among boys with hypospadias in Texas and Arkansas, but 26.3 times (95% CI, 23.1-29.9) more prevalent among boys with hypospadias in the NBDPN (heterogeneity *I*^2^ = 99.4%).

In addition, sensitivity analyses were performed using Texas data to assess whether the observed associations were associated with chromosomal anomalies. We repeated PR estimates after excluding individuals with any known chromosomal anomaly and found that syndromes caused by chromosomal anomalies explained a proportion of co-occurring hypospadias and CHDs (23% of all co-occurrences), but estimated PRs did not substantially differ in magnitude after these exclusions (eTable 4 in the Supplement).

### Analyses Among Only Boys With Hypospadias

We estimated that 7.024% of boys with hypospadias in Texas (95% CI, 7.020%-7.028%) and 5.503% of boys with hypospadias in Arkansas (95% CI, 5.495%-5.511%) have a co-occurring CHD (Table 3). We performed case-only analyses among boys with hypospadias to characterize risk factors for CHDs. We found that boys in Texas with third-degree (ie, more severe) hypospadias were 2.7 times (95% CI, 2.2-3.4) more likely than boys with first-degree hypospadias to have a co-occurring CHD, with consistent estimates in Arkansas (odds ratio, 2.7; 95% CI, 1.4-5.3). Likewise, we found that boys with hypospadias born to Hispanic mothers in Texas were 1.5 times (95% CI, 1.3-1.8) more likely than boys with hypospadias born to non-Hispanic White mothers to have a co-occurring CHD. The magnitude of association with Hispanic ethnicity was consistent in Arkansas (odds ratio, 1.6; 95% CI, 0.6-4.7) yet nonsignificant, likely owing to the low proportion of Hispanic individuals in the study population.

**Table 3.** Prevalence Estimates and Association Analyses of CHDs Only Among Boys With Hypospadias in the Texas Birth Defects Registry and Arkansas Reproductive Health Monitoring System

Factor	Texas Birth Defects Registry, 1999-2014				Arkansas Reproductive Health Monitoring System, 1995-2013			
	Boys, No.		Odds ratio (95% CI)^a^	*P* value	Boys, No.		Odds ratio (95% CI)^a^	*P* value
	With hypospadias	With hypospadias and CHDs			With hypospadias	With hypospadias and CHDs		
Any hypospadias	17 777	1343	7.024 (7.020-7.028)^b^	NA	2846	142	5.50 (5.49-5.51)^b^	NA
Hypospadias severity								
Not specified	7726	622	Excluded	NA	521	53	Excluded	NA
First degree	8240	520	1 [Reference]	NA	1577	60	1 [Reference]	NA
Second degree	1166	87	1.2 (1.0-1.5)	.11	628	16	0.5 (0.3-1.0)	.06
Third degree	645	114	2.7 (2.2-3.4)	<.001	120	13	2.7 (1.4-5.3)	<.003
Maternal race and ethnicity								
Hispanic	2927	282	1.5 (1.2-1.8)	<.001	77	8	1.6 (0.6-4.7)	.36
Non-Hispanic								
Black	1339	91	1.1 (0.8-1.4)	.62	357	22	1.0 (0.5-1.8)	.94
White	5314	319	1 [Reference]	NA	1941	109	1 [Reference]	NA
Other	471	29	1.0 (0.6-1.4)	.85	39	3	1.4 (0.3-6.1)	.64

Abbreviations: CHD, congenital heart defect; NA, not applicable.

^a^ Estimated from logistic regression models including hypospadias of specified severities (not specified severities excluded), maternal race and ethnicity, and birth year.

^b^ Prevalence of any CHD per 100 boys with hypospadias (with 95% CI). The prevalence estimates are among all boys with hypospadias, regardless of severity.

## Discussion

We analyzed 11 population-based birth defects registries that comprised more than 8.1 million pregnancies spanning 2 decades and found consistent evidence that boys with hypospadias are substantially more likely than boys without hypospadias to have a co-occurring major CHD. In general, boys with hypospadias have a 6-fold or greater risk for co-occurrence of any major CHD, with further elevated prevalence across specific CHDs. We found that boys with hypospadias have critical CHDs that require surgical intervention in the first years of life and have improved prognosis with earlier detection. In addition, our analyses suggest that differences in both hypospadias severity and maternal race and ethnicity could be associated with the likelihood for hypospadias to co-occur with a major CHD. Together, these findings suggest that, for a boy with a diagnosis of any form of hypospadias, there may be a benefit associated with screening for additional structural defects.

Screenings through prenatal sonography and newborn pulse oximetry are widely implemented to detect critical CHDs. Despite the success of these screening programs, these tests have imperfect sensitivity and positive predictive values for critical CHDs and often fail to detect noncritical CHDs.^35^ In addition, to our knowledge, there are currently no recommended additional screenings for boys with a diagnosis of hypospadias, regardless of hypospadias severity.^36^ However, it is increasingly recognized that boys with hypospadias could experience a benefit associated with clinical sequencing and genetic counseling,^19,37,38^ and the American College of Medical Genomics and Genetics now recommends sequencing for children with a diagnosis of a congenital anomaly prior to 1 year of age.^39^ Sequencing of individuals with multiple congenital anomalies, particularly those across body systems, has a high likelihood to yield a molecular diagnosis.^40,41^ Together, these findings suggest that additional clinical and genetic evaluations of boys with hypospadias may identify subclinical birth defects and improve molecular diagnoses for boys with multiple congenital anomalies.

Typically, defects that emerge in the same developmental fields are suspected to result from disturbances in shared, complex embryologic pathways.^42^ Hypospadias is known to frequently co-occur with other anomalies in the genitourinary system, likely owing to the shared developmental origins of the kidneys and urogenital tract.^43^ However, evidence is emerging that hypospadias frequently co-occurs with defects in other developmental fields. Agnostic analyses of co-occurring defects with hypospadias in the TBDR have established that hypospadias specifically clusters with CHDs beyond nonspecific birth defect clustering.^21^ Building on those findings using an expanded set of registry data, we found that 5.5% to 7.0% of boys with hypospadias had a major CHD, which is consistent with smaller hospital-based reports of developmental anomalies among boys treated for hypospadias (19 of 356 [5.3%] with CHD).^43^

The shared developmental etiology of urogenital and cardiac anomalies is now being investigated in molecular studies. In animal studies of single-gene CHDs, 29.8% of the variant lines exhibited renal anomalies.^44^ In addition, this study found that 30% of hospital-based patients with CHD exhibited kidney defects on imaging results, which together support the shared etiology between these systems. Genetic studies of children with CHDs revealed a complex architecture that includes single-gene disorders and polygenic inheritance.^45,46,47,48,49,50,51,52,53^ By contrast, less is known about the genetic etiology of hypospadias, but evidence is also emerging of a complex architecture.^54,55,56^ Approximately 70% of hypospadias cases occur outside of known genetic syndromes and have incompletely characterized origins.^57^ Many genetic syndromes that include hypospadias also feature CHDs, including Mowat-Wilson syndrome (*ZEB2* [OMIM 605802]),^58^ Opitz G/BBB syndrome (*MID1* [OMIM 300552]),^59^ and Wolf-Hirschhorn syndrome (*NSD2* [OMIM 602952], *LETM1* [OMIM 604407], and *MSX1* [OMIM 142983]).^60^

Risk for hypospadias and CHDs independently differs by maternal race or ethnicity.^3,61,62,63^ We found that boys with hypospadias born to Hispanic mothers were more likely to have a co-occurring CHD than boys with hypospadias born to non-Hispanic White mothers. We additionally identified a higher prevalence of CHDs among boys with hypospadias in Texas, which may reflect the high proportion of Hispanic individuals living in Texas compared with Arkansas. Disparities in outcomes for select CHDs disproportionately affect Hispanic individuals,^64^ so it is important to identify vulnerable populations that could benefit from enhanced screening and surveillance of co-occurring conditions.

### Strengths and Limitations

Our study has some strengths. We performed independent analyses of 3 birth defect databases comprising 11 states. Prevalence of specific CHDs in association with hypospadias was estimated in 2 states with active surveillance programs, which (1) minimizes information bias owing to differences in surveillance programs, (2) is a powerful approach to identify rare co-occurring birth defects, and (3) minimizes sampling bias owing to the large population-based samples. In addition, a large proportion of cases in Texas (60%) and Arkansas (30%) are reviewed by a clinical geneticist. We also used a large, multistate network of surveillance programs to statistically validate findings in our primary analyses.

However, our study should be considered in light of certain limitations. Our approach did not account for the known tendency of birth defects to cluster nonspecifically,^65^ which makes it difficult to make inferences about the observed associations relative to associations between other birth defects. That is, we expect that many pairwise combinations of birth defects will have an increased association even without shared etiology, but the magnitude of association at which shared etiology is suggested remains unknown. In addition, birth defect registries are limited to major defects reportable to each registry. Specific CHDs that are considered minor defects (those with less morbidity or mortality and incomplete ascertainment) are therefore not included in these registry analyses, so we cannot assess from these analyses whether prevalence of minor CHDs was also elevated with hypospadias. Likewise, minor and noncritical CHDs may go undiagnosed early in life, yet they may become symptomatic as the individual ages, so it is important to evaluate whether these defects are also more likely to co-occur with hypospadias. We found that atrial septal defects and ventricular septal defects—which may go undiagnosed—are more prevalent with hypospadias but had estimates that differed between the surveillance systems. These CHDs may be incorrectly captured through birth defect registries, so it is difficult to know whether the prevalence estimates reported for atrial septal defects and ventricular septal defects are accurate.

## Conclusions

The high prevalence of co-occurring CHDs in this cohort study suggests that hypospadias may not be an isolated defect and that additional birth defect screening among boys born with hypospadias is warranted. The ability to characterize molecular alterations associated with the development of both hypospadias and CHDs may lead to targeted prevention strategies and improved counseling for these relatively common and medically significant birth defects.

## Notes

Supplement. **eTable 1.** British Pediatric Association (BPA) Codes Used for the Identification of Congenital Heart Defects (CHDs) in Each Registry **eTable 2.** Description of Boys From Nine States in the National Birth Defects Prevention Network, 1999-2007 **eTable 3.** Meta-Analyses of Hypospadias-CHD Prevalence Ratios Among Males in Texas (TBDR, 1999-2014), Arkansas (ARHMS, 1995-2013), and the National Birth Defects Prevention Network (NBDPN, 1999-2007) **eTable 4.** Sensitivity Analyses Among Males in the Texas Birth Defects Registry (TBDR, 1999-2014) Excluding Individuals With Any Chromosomal Anomaly Identified by British Pediatric Association Coding
