# Supplementary material for: Prevalence and Clustering of Congenital Heart Defects Among Boys With Hypospadias
Source: JAMA Netw Open. 2022 Jul 28;5(7):e2224152. doi: 10.1001/jamanetworkopen.2022.24152 (PMC9335139; doi:10.1001/jamanetworkopen.2022.24152)
Supplement: Supplement. — eTable 1. British Pediatric Association (BPA) Codes Used for the Identification of Congenital Heart Defects (CHDs) in Each Registry eTable 2. Description of Boys From Nine States in the National Birth Defects Prevention Network, 1999-2007 eTable 3. Meta-Analyses of Hypospadias-CHD Prevalence Ratios Among Males in Texas (TBDR, 1999-2014), Arkansas (ARHMS, 1995-2013), and the National Birth Defects Prevention Network (NBDPN, 1999-2007) eTable 4. Sensitivity Analyses Among Males in the Texas Birth Defects Registry (TBDR, 1999-2014) Excluding Individuals With Any Chromosomal Anomaly Identified by British Pediatric Association Coding [file jamanetwopen-e2224152-s001.pdf]

Supplementary Online Content

Richard MA, Patel J, Benjamin RH, et al. Prevalence and clustering of congenital heart defects among boys with hypospadias. *JAMA Netw Open.* 2022;5(7):e2224152. doi:10.1001/jamanetworkopen.2022.24152

eTable 1. British Pediatric Association (BPA) Codes Used for the Identification of Congenital Heart Defects (CHDs) in Each Registry
eTable 2. Description of Boys From Nine States in the National Birth Defects Prevention Network, 1999-2007
eTable 3. Meta-Analyses of Hypospadias-CHD Prevalence Ratios Among Males in Texas (TBDR, 1999-2014), Arkansas (ARHMS, 1995-2013), and the National Birth Defects Prevention Network (NBDPN, 1999-2007)
eTable 4. Sensitivity Analyses Among Males in the Texas Birth Defects Registry (TBDR, 1999-2014) Excluding Individuals With Any Chromosomal Anomaly Identified by British Pediatric Association Coding

This supplementary material has been provided by the authors to give readers additional information about their work.

© 2022 Richard MA et al. *JAMA Network Open.*

eTable 1. British Pediatric Association (BPA) Codes Used for the Identification of Congenital Heart Defects (CHDs) in Each Registry

CHD category	BPA group	BPA codes included	BPA codes excluded	Critical CHD
**All CHD**	**745, 746, 747**	**all 745, 746, 747 codes below**		
**Bulbus Cordis Anomalies and Anomalies of Cardiac Septal Closure**	**745**	**all 745 codes below**		
Common truncus arteriosus	7450	745000, 745010		Yes
Transposition of the great vessels	7451	745100, 745110, 745120, 745130, 745140, 745150, 745180, 745190		Yes
Tetralogy of Fallot	7452	745200, 745210, 746840		Yes
Single ventricle	7453	745300		Yes
Ventricular septal defect	7454	745400, 745410, 745420, 745480, 745485, 745486, 745487, 745490, 745498		
Atrial septal defect	7455	745510, 745520, 745580, 745590	745500	
Atrioventricular septal defect	7456	745600, 745610, 745620, 745630, 745680, 745690		
Cor biloculare	7457	745700		
Other specified defects of septal closure	7458	745800		
Unspecified defect of septal closure	7459	745900		
**Other Congenital Anomalies of Heart**	**746**	**all 746 codes below**		
Pulmonary valve atresia or stenosis	7460	746000, 746010, 746090	746020, 746080	
Tricuspid valve atresia or stenosis	7461	746100, 746106	746105	
Ebstein anomaly	7462	746200		Yes
Aortic valve stenosis	7463	746300		
Congenital insufficiency of aortic valve	7464	746470, 746490	746400, 746480	
Congenital mitral stenosis	7465	746500, 746505		
Mitral valve insufficiency	7466	746600	746600	
Hypoplastic left heart syndrome	7467	746700		Yes
Other specified anomalies of the heart	7468	746810, 746820, 746830, 746840, 746850, 746870, 746880, 746881, 746882, 746883, 746885, 746887	746800, 746860, 746886	
Unspecified anomalies of heart (excluding 746900, 746990)	7469	746910, 746920, 746930, 746995	746900, 746990	
**Other Congenital Anomalies of Circulatory System**	**747**	**all 747 codes below**		
Patent ductus arteriosus	7470	747008	747000	
Coarctation of the aorta	7471	747100, 747110, 747190		Yes
Other anomalies of aorta	7472	747200, 747210, 747215, 747216, 747217, 747220, 747230, 747240, 747250, 747260, 747270, 747280, 747285, 747290		

© 2022 Richard MA et al. *JAMA Network Open.*

Anomalies of pulmonary artery	7473	747300, 747310, 747320, 747330, 747340, 747380, 747390	747325	
Anomalies of great veins	7474	747400, 747410, 747420, 747430, 747440, 747450, 747480, 747490		

© 2022 Richard MA et al. *JAMA Network Open.*

eTable 2. Description of Boys From Nine States in the National Birth Defects Prevention Network, 1999-2007

	**National Birth Defects Prevention Network 1999-2007**							
	**N=4,404,236**							
	Boys with CHD		Boys with hypospadias		Boys with hypospadias and CHD		Boys without birth defects	
	n	%	n	%	n	%	n	%
**Maternal race/ethnicity**								
Hispanic	2,209	20.7	1,172	9.6	64	11.8	955,068	21.8
Non-Hispanic Black	1,596	14.9	1,796	14.6	115	21.1	699,589	16.0
Non-Hispanic White	6,174	57.7	8,695	70.9	341	62.7	2,422,958	55.3
Other[A]	712	6.7	602	4.9	24	4.4	303,121	6.9
**Birth year**								
1999	772	7.2	885	7.2	42	7.7	400,282	9.1
2000	944	8.8	1,049	8.6	42	7.7	450,965	10.3
2001	923	8.6	872	7.1	25	4.6	449,669	10.3
2002	1,069	10.0	1,124	9.2	58	10.7	449,299	10.3
2003	1,384	12.9	1,740	14.2	65	11.9	517,732	11.8
2004	1,423	13.3	1,835	15.0	85	15.6	516,899	11.8
2005	1,381	12.9	1,571	12.8	70	12.9	523,196	11.9
2006	1,396	13.1	1,587	12.9	83	15.3	531,894	12.1
2007	1,399	13.1	1,602	13.1	74	13.6	540,800	12.3

A, Other race/ethnicities may include individuals who report unknown race, multiple races, East Asian, South Asian, Native American, Alaskan Native, or Native Hawaiian ancestry.

© 2022 Richard MA et al. *JAMA Network Open.*

eTable 3. Meta-Analyses of Hypospadias-CHD Prevalence Ratios Among Males in Texas (TBDR, 1999-2014), Arkansas (ARHMS, 1995-2013), and the National Birth Defects Prevention Network (NBDPN, 1999-2007)

	**Single-State Registry Analyses**		**National Birth Defects Prevention Network N=4,404,236**			**Meta-Analysis[B] TBDR, ARHMS, and NBDPN**	
	TBDR PR	ARHMS PR	Boys with hypospadias and CHD n	Boys with CHD n	PR[A] (95% CI)	PR (95% CI)	Test for Heterogeneity[C] I^2
**All CHDs**	**5.8**	**5.8**	**544**	**10,691**	**16.1 (14.7, 17.6)**	**7.6 (7.3, 7.9)**	**99.5**
**Bulbus Cordis Anomalies and Anomalies of Cardiac Septal Closure**	**5.8**	**6.4**	**458**	**7,927**	**17.5 (15.9, 19.2)**	**7.7 (7.4, 8.1)**	**99.5**
Common truncus arteriosus	9.4	-	6	269	7.3 (3.2, 16.6)	8.7 (5.5, 13.8)	0
Transposition of the great vessels	6.1	8	25	1,752	5.0 (3.4, 7.5)	5.9 (4.9, 7.2)	0
Tetralogy of Fallot	9.5	7.1	37	1,708	7.7 (5.5, 10.7)	8.7 (7.2, 10.5)	0
Single ventricle	2.8	-	4	140	6.4 (2.3, 17.3)	4.0 (2.1, 7.8)	32.1
Ventricular septal defect	6.1	6.7	270	2,652	26.3 (23.1, 29.9)	9.1 (8.6, 9.8)	99.4
Atrial septal defect	6	7.1	194	2,532	19.1 (16.5, 22.2)	7.6 (7.1, 8.1)	98.9
Atrioventricular septal defect	5.7	7.2	18	1,257	4.9 (3.1, 7.9)	5.6 (4.4, 7.1)	0
**Other Congenital Anomalies of Heart**	**6.2**	**4.6**	**132**	**3,026**	**12.8 (10.8, 15.3)**	**7.4 (6.7, 8.1)**	**96.3**
Pulmonary valve atresia or stenosis	5.5	3.6	46	651	19.5 (14.4, 26.4)	7.9 (6.7, 9.4)	96.0
Tricuspid valve atresia or stenosis	6.1	-	7	153	11.7 (5.5, 25.1)	7.2 (4.9, 10.5)	53.0
Ebstein anomaly	2.8	-	2	70	-	2.8 (1.0, 7.5)	0
Aortic valve stenosis	5.9	4.9	12	662	7.0 (3.9, 12.4)	6.0 (4.6, 8.0)	0
Congenital insufficiency of aortic valve	8	-	8	54	30.6 (14.4, 64.7)	8.8 (7.2, 10.9)	91.2
Congenital mitral stenosis	6.7	6.6	6	247	6.0 (2.6, 13.5)	6.6 (5.1, 8.5)	0
Mitral valve insufficiency	9.8	-	41	381	22.7 (16.4, 31.5)	11.0 (9.7, 12.4)	95.4
Hypoplastic left heart syndrome	6.1	-	19	1,014	6.2 (3.9, 9.8)	6.1 (4.6, 8.1)	0
Other specified anomalies of the heart	6.1	7.6	45	649	15.1 (11.1, 20.4)	7.8 (6.7, 9.2)	96.0
Unspecified anomalies of heart	6.4	12.8	10	188	12.6 (6.7, 23.9)	8.6 (6.2, 11.9)	51.6

© 2022 Richard MA et al. *JAMA Network Open.*

**Other Congenital Anomalies of Circulatory System**	**7.8**	**6.7**	**126**	**3,372**	**11.3 (9.4, 13.5)**	**8.4 (7.8, 9.2)**	**85.1**
Coarctation of the aorta	7.3	9	40	2,059	6.7 (4.9, 9.2)	7.3 (6.1, 8.6)	0
Other anomalies of aorta	8.6	7.1	37	810	11.1 (7.9, 15.4)	8.8 (7.8, 9.9)	22.3
Anomalies of pulmonary artery	6.6	-	40	656	15 (10.9, 20.7)	8.2 (7.0, 9.7)	94.5
Anomalies of great veins	9.5	3.4	38	400	22.7 (16.2, 31.9)	11.4 (9.7, 13.4)	91.7

A, Poisson regression models in NBDPN are adjusted for maternal race/ethnicity, birth year, and state
B, Adjusted prevalence ratios from TBDR, ARHMS, and NBDPN were combined using fixed effects inverse-variance weighted meta-analyses.
C, $I^2<60\%$ considered homogeneous effects between studies, $I^2\geq80\%$ considered substantial heterogeneity of effects between studies.

© 2022 Richard MA et al. *JAMA Network Open.*

eTable 4. Sensitivity Analyses Among Males in the Texas Birth Defects Registry (TBDR, 1999-2014)[A] Excluding Individuals With Any Chromosomal Anomaly Identified by British Pediatric Association Coding

	Including chromosomal anomalies			Excluding chromosomal anomalies		
	Boys with hypospadias and CHD n	Boys with CHD n	PR[B] (95% CI)	Boys with hypospadias and CHD n	Boys with CHD n	PR[B] (95% CI)
**All CHD**	**1,343**	**38,691**	**5.8 (5.5, 6.2)**	**1,040**	**34,356**	**5.4 (5.1, 5.7)**
**Bulbus Cordis Anomalies and Anomalies of Cardiac Septal Closure**	**1,155**	**33,283**	**5.8 (5.5, 6.2)**	**883**	**29,383**	**5.4 (5.0, 5.7)**
Common truncus arteriosus[C]	13	238	9.4 (5.4, 16.4)	8	191	9.2 (4.9, 17.5)
Transposition of the great vessels[C]	70	1,943	6.1 (4.8, 7.7)	58	1,800	5.7 (4.4, 7.4)
Tetralogy of Fallot[C]	71	1,217	9.5 (7.5, 12.1)	52	917	9.6 (7.3, 12.6)
Single ventricle[C]	5	327	2.8 (1.2, 6.8)	<5	308	-
Ventricular septal defect	599	16,811	6.1 (5.7, 6.7)	446	15,067	5.4 (4.9, 5.9)
Atrial septal defect	697	19,281	6.0 (5.6, 6.5)	534	16,817	5.6 (5.2, 6.1)
Atrioventricular septal defect	46	1,322	5.7 (4.3, 7.7)	22	651	6.3 (4.2, 9.4)
**Other Congenital Anomalies of Heart**	**332**	**8,891**	**6.2 (5.6, 7.0)**	**235**	**7,780**	**5.3 (4.6, 6.0)**
Pulmonary valve atresia or stenosis	88	2,715	5.5 (4.5, 6.8)	72	2,504	5.1 (4.0, 6.4)
Tricuspid valve atresia or stenosis	20	555	6.1 (3.9, 9.5)	12	491	4.2 (2.4, 7.4)
Ebstein anomaly[C]	<5	240	2.8 (1.0, 7.5)	<5	222	3.1 (1.2, 8.4)
Aortic valve stenosis	34	938	5.9 (4.2, 8.3)	27	885	5.4 (3.7, 7.8)
Congenital insufficiency of aortic valve	85	1,764	8.0 (6.4, 9.9)	56	1,507	6.3 (4.8, 8.2)
Congenital mitral stenosis	53	1,297	6.7 (5.1, 8.8)	35	1,053	5.9 (4.3, 8.2)
Mitral valve insufficiency	242	4,224	9.8 (8.6, 11.2)	202	3,736	9.6 (8.3, 11.0)
Hypoplastic left heart syndrome[C]	32	844	6.1 (4.3, 8.7)	25	771	5.3 (3.5, 7.8)
Other specified anomalies of the heart	110	3,079	6.1 (5.0, 7.3)	80	2,688	5.3 (4.3, 6.6)
Unspecified anomalies of heart	22	578	6.4 (4.2, 9.9)	11	518	3.9 (2.2, 7.0)
**Other Congenital Anomalies of Circulatory System**	**407**	**8,744**	**7.8 (7.1, 8.7)**	**301**	**7,620**	**7.1 (6.4, 8.0)**
Coarctation of the aorta[C]	85	1,935	7.3 (5.9, 9.1)	65	1,759	6.4 (5.0, 8.2)
Other anomalies of aorta	231	4,496	8.6 (7.5, 9.8)	171	3,816	8.0 (6.9, 9.3)
Anomalies of pulmonary artery	107	2,764	6.6 (5.5, 8.0)	82	2,451	6.2 (5.1, 7.7)

© 2022 Richard MA et al. *JAMA Network Open.*

Anomalies of great veins	116	2,113	9.5 (7.9, 11.4)	80	1,805	8.5 (6.9, 10.5)

A, Counts less than five have been suppressed per data use agreements with the TBDR.

B, Poisson regression models are adjusted for maternal race/ethnicity and birth year.

C, Critical congenital heart defect.

© 2022 Richard MA et al. *JAMA Network Open.*
